# Fragmentation of microspheres after bronchial artery injection: a case report and review of the literature

**DOI:** 10.1186/s13256-021-03099-4

**Published:** 2021-10-26

**Authors:** Wei Luo, Tongchen Hu, Yu Mao, Qi Yu

**Affiliations:** 1 Department of Respiratory and Critical Care Medicine, The People’s Hospital of Leshan, Leshan, 614000 Sichuan People’s Republic of China; 2 Department of Thoracic Surgery, The People’s Hospital of Leshan, Leshan, 614000 Sichuan People’s Republic of China; 3 Department of Pathology, The People’s Hospital of Leshan, Leshan, 614000 Sichuan People’s Republic of China; 4 Department of Pediatric Medicine, The People’s Hospital of Leshan, Leshan, 614000 Sichuan People’s Republic of China

**Keywords:** Aspergillomas, Massive hemoptysis, Embolization, Polyvinyl alcohol microspheres, Case report

## Abstract

**Background:**

Massive hemoptysis due to aspergilloma is a rare but life-threatening complication. Bronchial artery embolization is recommended as a definitive treatment for massive hemoptysis. Polyvinyl alcohol is widely used in bronchial artery embolization. A very small number of studies have reported disrupted polyvinyl alcohol, which may cause ectopic embolism.

**Case presentation:**

This case highlights an unusual phenomenon in which polyvinyl alcohol fragments appeared on pathological examination in a 61-year-old man, ethnic Han, with massive hemoptysis caused by aspergilloma for whom bronchial artery embolization failed. Lobectomy was carried out successfully. Hematoxylin and eosin stain provides clear images of polyvinyl alcohol fragments, while alpha-smooth muscle cell actin and cluster of differentiation-34 immunohistochemistry revealed their localization in bronchioles.

**Conclusion:**

Thus far, only two cases of polyvinyl alcohol fragments in the lung have been reported, and the mechanism has not been elucidated. These two cases revealed no counter-indication for the use of polyvinyl alcohol. However, in some cases of off-target embolization causing fatal complications, such as stroke, paraplegia, and myocardial, polyvinyl alcohol fragmentation needs to be taken into consideration.

## Introduction

Aspergillomas are mass-like fungus balls that are typically composed of *Aspergillus fumigatus*, most of which are secondary to structurally abnormal lungs, especially those with preexisting cavities. Their main clinical features are recurrent hemoptysis and different amounts of hemoptysis [1]. Massive hemoptysis due to aspergilloma is a rare but deadly complication, with an estimated mortality as high as 38% [2]. Bronchial artery embolization (BAE) is recommended as a temporary measure before surgery, or as a definitive treatment for massive hemoptysis [2]. Polyvinyl alcohol (PVA) is widely used in BAE for its permanent embolization effect. A very small number of studies have reported disrupted PVA, which may cause ectopic embolism [3].

This case provides unique and clear images of PVA fragments in the lung specimen, which probably provide a new explanation for ectopic embolism.

## Case presentation

**Fig. 1:**
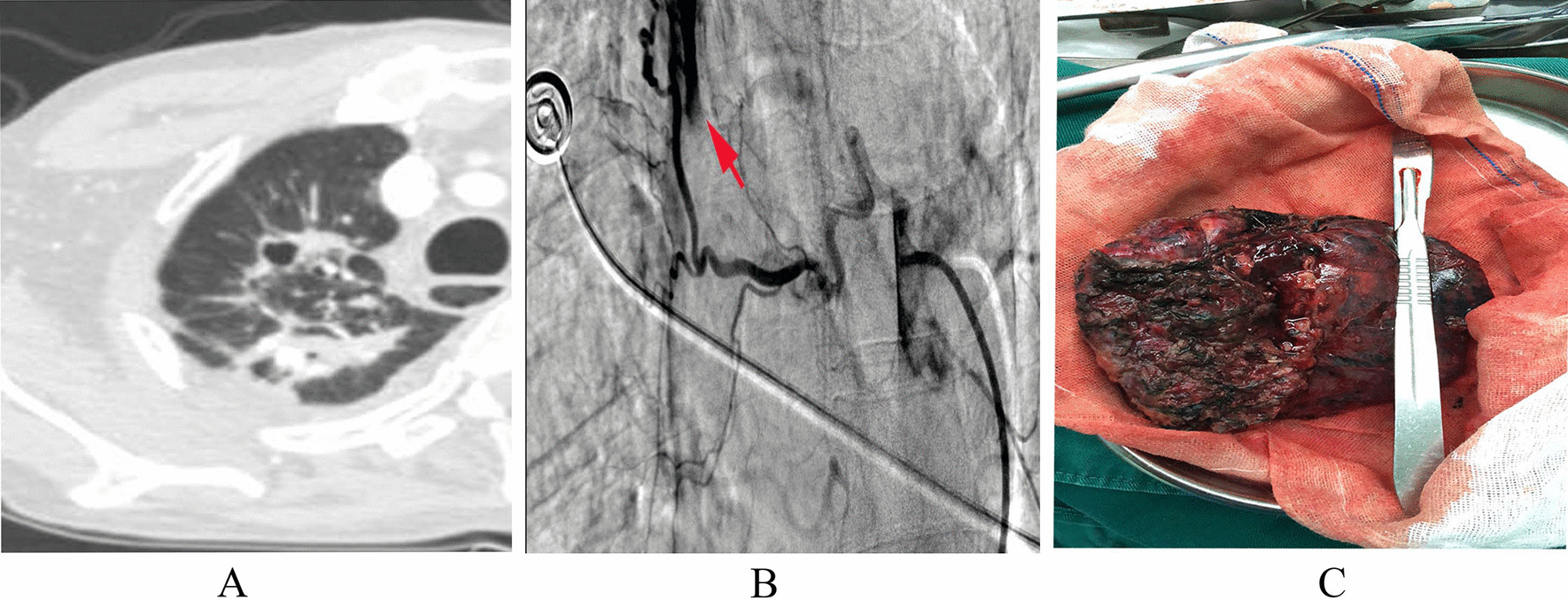
**a** Chest computerized tomography scan showing bilateral apical post-tuberculosis lung fibrosis and a right apical 2.5 × 2 cm thick-walled cavity with a solid intracavity mass bearing the air crescent sign. **b** Right bronchial artery angiography showing tortuosity, hypertrophy, and extravasation of contrast material into the right upper-lobe bronchus (red arrow). **c** Gross pathologic specimen after surgical resection of the right lobe

**Fig. 2:**
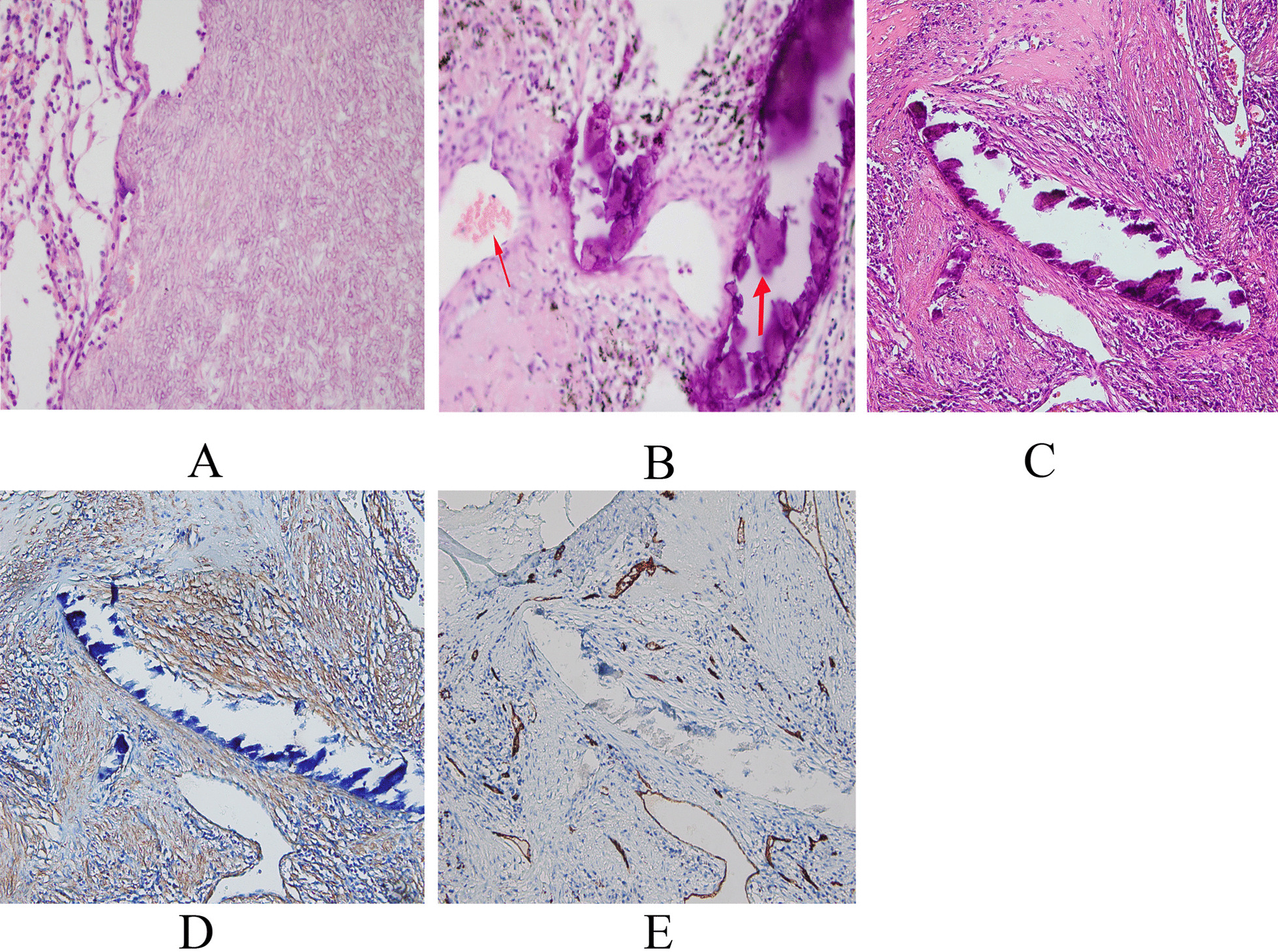
**a** Septate hyphae in a resected cavity with a chronic inflammatory reaction. Hematoxylin and eosin (H&E) staining, original magnification ×400. **b** Basophilic-appearing PVA fragment (thick arrow) and red blood cells in the lung (thin arrow). H&E staining, original magnification ×400. **c** Basophilic-appearing PVA fragment. H&E staining, original magnification ×200. **d** Alpha-SMA immunohistochemistry showing the fragments were not in the vessel but in the bronchioles, original magnification ×200. **e** CD34 immunohistochemistry showing the fragments were not in the vessel but in the bronchioles, original magnification ×200

**Fig. 3:**
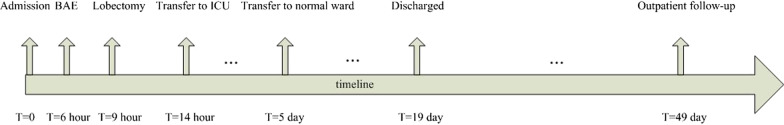
Timeline demonstrating important dates for the patient in hospital and on outpatient follow-up

Written consent was obtained from our institutional review board and the patient for this case report as well as accompanying images. A 61-year-old man, ethnic Han, presented with massive hemoptysis of nearly 500 mL of fresh blood. He did not complain of any ongoing respiratory symptoms. His past medical history included pulmonary tuberculosis (TB) with 6 months of standard antituberculosis therapy (2HRZE/4HR, 2HRZE: isoniazid 300 mg once daily, rifampin 450 mg once daily, pyrazinamide 750 mg twice daily and ethambutol 750 mg once daily for 2 months; 4HR: isoniazid 300 mg once daily plus rifampin 450 mg once daily for 4 months), and outpatient follow-up showed resolution of his TB. This patient was a farmer, while social, environmental, family, and psychosocial history was unremarkable. He did not smoke or consume alcohol. The patient had a respiratory rate of 30 breaths/minute and oxygen saturation of 92% on ambient air. Chest physical examination revealed mild respiratory distress, that is, decreased breath sounds on the top right side of the chest. Other physical examination was unremarkable. Chest computerized tomography scan showed bilateral apical post-tuberculosis lung fibrosis and a right apical 2.5 × 2 cm^2^ thick-walled cavity with a solid intracavity mass bearing the air crescent sign, while enhanced computerized tomography (CT) scan indicated remarkable enhancement around the lesion and no obvious fistula. (Fig. 1a). BAE was carried out, and digitally subtracted angiography demonstrated that the right bronchial arteries were abnormal, with tortuosity, hypertrophy, and extravasation of contrast material into the right bronchus (Fig. 1b). Because of the tortuosity of bronchial artery, the microcatheter could not be reliably and stably imported; thus, steel platinum coils were not an option. One gram of PVA microspheres (Hegui, China) with a diameter of 700–900 µm were chosen to embolize the culprit bronchial artery. However, the embolic agents appeared quickly in the right upper-lobe bronchus after slow and gentle injection into the bronchial artery. Rapid deterioration during the procedure, including ongoing hemoptysis, tachycardia, and hypotension, necessitated surgical resection of the right upper lobe (Fig. 1c). Pathological examination demonstrated not only septate hyphae in a resected cavity with a chronic inflammatory reaction (Fig. 2a) but also a basophilic-appearing PVA fragment in the lung (Fig. 2b, c). After several days of stay in the intensive care unit, the patient, lacking any symptoms of hemoptysis and ectopic embolism, was transferred to a normal ward and discharged 2 weeks later. This patient received 1600 mL suspended erythrocyte and 1600 mL plasma transfusion during hospitalization. One month later, outpatient follow-up showed good recovery except a little bit of right chest pain. Figure 3 is a timeline demonstrating the important dates for the patient in hospital and on outpatient follow-up.

## Discussion

PVA has been widely used in BAE for treatment of massive hemoptysis for its permanent embolization effect and relative easy drug-delivery access (no need for microcatheter) compared with gelatin sponge particles and stainless steel platinum coils. In this case, PVA was chosen in the hope of occluding the fistulas. With the accumulation of PVA with contrast material, the shape of the right main bronchi was clear; therefore, surgical resection was carried out. Pathologic examination demonstrated PVA fragments. Alpha-smooth muscle cell actin (Alpha-SMA) and CD34 immunohistochemistry was carried to locate the fragments. To the best of our knowledge, few cases have reported images of disrupted PVA in the human lung [3, 4].

A systematic search of MEDLINE and EMBASE was conducted from inception to 25 July 2021, using the search terms “PVA AND bronchial artery embolism,” “massive hemoptysis AND bronchial artery embolism,” “massive hemoptysis AND PVA AND bronchial artery embolism,” “massive hemoptysis AND aspergillomas AND PVA AND Bronchial artery embolism.” Only two reports were found (Table 1). Robbins and colleagues reported that microsphere fragments appeared in the lung vessels, while Bonnefoy *et al*. also captured images of particles in the lungs [4, 5].

**Table 1:** Reports of microsphere images in the lung

Publishing year	Patient’s disease	Receive BAE	Embolic agent	Emerging PVA fragments
2015	Chronic pulmonary thromboembolic disease	Yes	PVA	Yes
2018	Bronchial Dieulafoy disease	Yes	PVA	Not sure
Present case	Aspergillomas	Yes	PVA	Yes

*PVA* Polyvinyl alcohol

As can be seen in Fig. 2d, e, the fragments were not in the vessel but in the bronchioles. This could be explained by the fistula between bronchial artery and the bronchioles. Of interest to us was the size of the PVA fragments, which scattered around the bronchioles with different diameters. Some of the fragments were just as large as the red blood cells (Fig. 2b). This phenomenon raises the question of whether the PVA fragmentation occurred during specimen preparation or in the human body or associating with TB or *Aspergillus*. For the first possibility, our slice thickness was 5 µm, while PVA exceeding this thickness would be expected to break during histological preparation. We are more curious about the latter possibility. As Fig. 2b, c shows, the contour of the PVA fragments is not clear and lacks a cutting edge, while many small fragments of different sizes are scattered in the vessel. The mechanism of PVA fragmentation *in vivo* was not clear, though it may be related to the mechanical force of injection. However, we used a 5F Cobra angiography catheter (Terumo, Japan) with 1.65 mm inner diameter, which is much larger than 900 µm of largest PVA size, so this probability is very low. We could exclude the possibility that PVA fragmentation was associated with TB or *Aspergillus* according to the pathological features. The PVA fragmentation appeared in the bronchioles, and this is cause for great concern, because embolic agents smaller than 50 µm might pass through the physiological arteriovenous shunt to the systemic arteries, resulting in ectopic embolisms [6]. In fact, there are several reported cases of off-target embolization causing stroke, though we were not convinced by some of the proposed mechanisms. In these cases, there was no presence of collateral circulation, no visible shunt, and no known mechanisms proposed by Knight [7], but the strokes happened after BAE [8, 9]. The authors hypothesized that the microspheres probably passed through an unvisualized right-to-left shunt from the right pulmonary arteries to the right pulmonary veins or created a thrombus during the procedure, dropping into the vertebral artery and causing an embolic stroke [8, 9]. Moreover, we also observed a stroke during the procedure of drug-eluting bead bronchial arterial chemoembolization in a lung cancer patient. Although we proposed that mechanical forces disrupted the unvisualized anastomoses, which opened errant emboli passages through the pulmonary vein and allowed off-target embolization of the intracranial arteries [10], we could not rule out the possibility of PVA fragmentation.

## Conclusions

This case raised a concern about the safety of PVA when applied in humans. Thus far, only two cases have reported the fragmentation of PVA, and the mechanism has not been elucidated. We could not make any conclusion based on the two cases. Moreover, there are tens of thousands of cases treated successfully with PVA every year, so this does not contradict the use of PVA. However, in some cases of off-target embolization causing fatal complication, such as stroke, paraplegia, and myocardial, PVA fragmentation needs to be taken into consideration.

## Data Availability

All data and materials are available.

## Abbreviations

BAE: Bronchial artery embolization
PVA: Polyvinyl alcohol
TB: Tuberculosis
CT: Computerized tomography
Alpha-SMA: Alpha-smooth muscle cell actin
